# A new tool for annotating scientific animations and supporting scientific dialogue

**DOI:** 10.1371/journal.pbio.3001731

**Published:** 2022-08-04

**Authors:** Hui Liu, Margot Riggi, Jen Rogers, Miriah Meyer, Janet H. Iwasa

**Affiliations:** 1 Art & Science Research Center, University of Science and Technology of China, Hefei, Anhui, China; 2 Department of Biochemistry, University of Utah, Salt Lake City, Utah, United States of America; 3 SCI Institute, School of Computing, University of Utah, Salt Lake City, Utah, United States of America; 4 Department of Science and Technology, Linköping University, Norrköping, Sweden

## Abstract

A new tool for annotating scientific animations enables users to interactively explore the data used to create the animation and engage in scientific discourse through comments and questions. Iwasa and colleagues demonstrate its power with an animation of the life cycle of SARS-CoV-2.

## Teaser

A new interactive annotation interface supports a detailed molecular animation of the SARS-CoV-2 life cycle. With this tool, users can interactively explore the data used to create the animation and engage in scientific discourse through comments and questions.

## A growing role for 3D molecular animations within the scientific community

The constant and rapid influx of new data during the Severe Acute Respiratory Syndrome Coronavirus 2 (SARS-CoV-2) pandemic highlighted the need for accessible, curated, and synthesized information about ongoing biological research. 3D animation is an ideal technique to integrate complex and often disparate scientific data into seamless and engaging stories. Beyond the well-accepted roles of polished animations in education and communication, the iterative creative process also has a part to play in the way scientists conceive of, discuss, and understand complex and dynamic processes. Developing and revising scientific animations can thus help build consensus models. Animations can capture diverse biological activities with far more detail and precision than words, an important consideration when attempting to describe and distinguish hypotheses of events that occur on a nanoscale level [1–3]. However, scientific animations have a significant and inherent weakness: They lack transparency. From watching an animation, it is difficult to differentiate between segments that are well grounded in experimental evidence and those that are based largely on speculation. In addition, while peer-reviewed scientific articles typically end with an extensive reference list that is instrumental in providing background knowledge, acknowledging the contributions from others, and supporting the authors’ hypotheses, there is currently no established system to describe and share the references similarly used during the creation of an animation [3–4]. Adding to the complexity of the issue is the wide variety of sources that animators may use, including personal communications with experts, database queries, images, simulations, videos, structural files, and research articles. This lack of annotation can damage the credibility of scientific animations and justifiably cause skepticism within the research community [5]. As animation becomes more prevalent as a means of scientific and public communication, it has become increasingly important to consider methods that increase the transparency of animations, both to describe creative choices made by the animator, as well as the variety of data sources (or the lack of data) that have informed them.

This project takes a novel approach for enabling transparent communication of scientific knowledge within the scientific research community and beyond. We have chosen to develop a visualization of the SARS-CoV-2 life cycle to demonstrate how a detailed 3D molecular animation can be integrated into a novel user interface that enables annotation and support for scientific discourse.

## Developing an animated model of the SARS-CoV-2 life cycle

**Fig 1:**
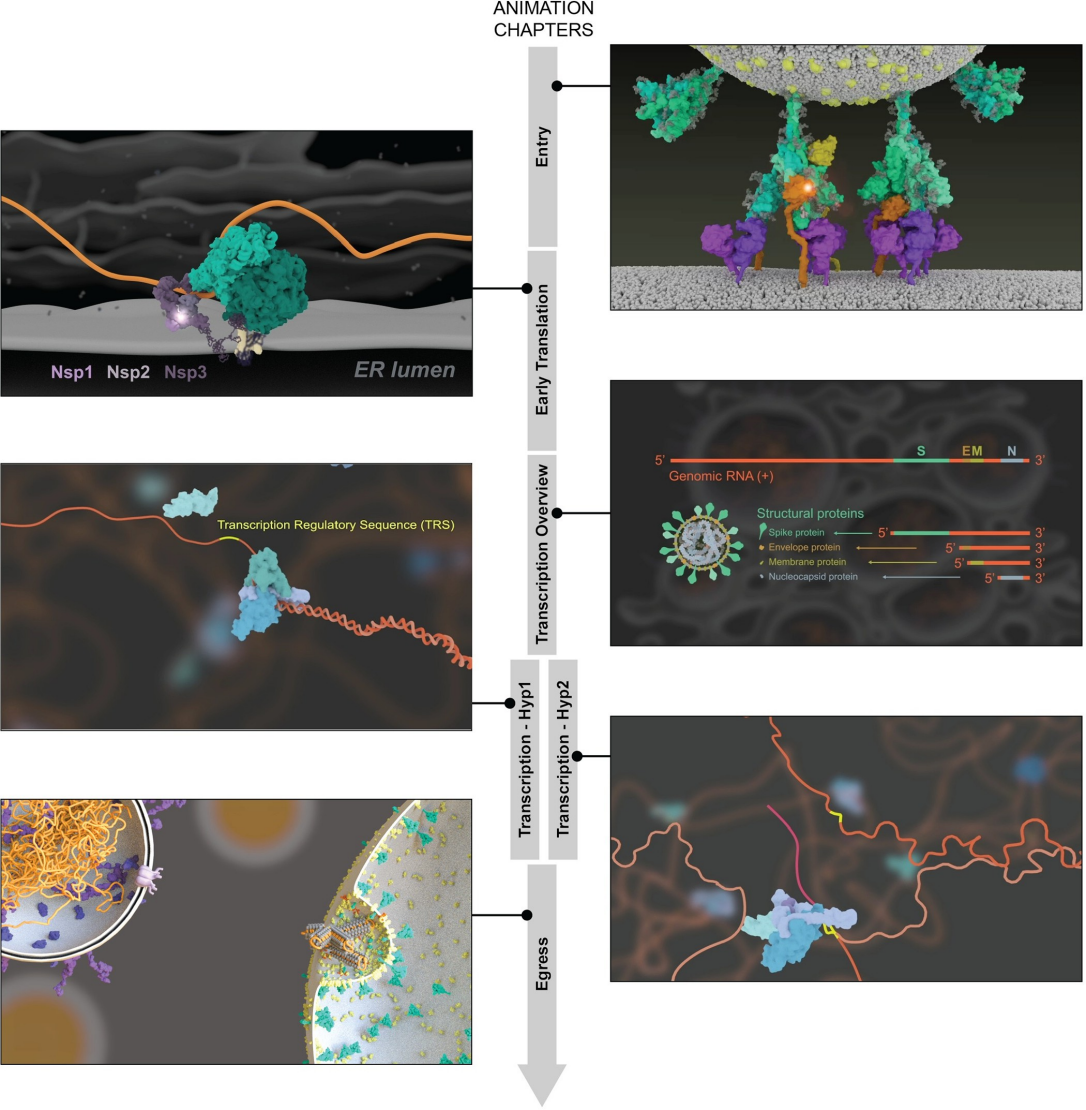
Representative still images from each of the chapters of the SARS-CoV-2 life cycle animation. Visit https://coronavirus-annotation-3.sci.utah.edu/ to watch the animation that is embedded in the annotation and commenting tool. If you’d like to download the animation or view it outside of the tool, you can do so on Vimeo: https://vimeo.com/510310488.

We created an animation that describes the full life cycle of SARS-CoV-2 (Fig 1), following a standard procedure that involves consulting with scientific experts [6], and that was planned to be iteratively revised based on community feedback. This animation does not discuss potential differences between variants, nor does it cover virus/host interactions or the inflammatory response to the infection.

When we initiated this project at the onset of the pandemic, there was still limited information available in the published literature on the molecular basis of SARS-CoV-2 infection. Although many research groups were making their findings quickly accessible online, this created a significant challenge for us: How do we show which aspects of SARS-CoV-2 biology were already well grounded in experimental evidence, and which areas still represented “black boxes” lacking adequate research coverage? How might we visualize competing hypotheses? Is it possible to reconcile the current knowledge into a single, linear, animated consensus model?

While the mechanism of SARS-CoV-2 entry into the host cell was relatively well understood, much was still unknown about the latter stages of the life cycle. One major point of uncertainty was the formation of double-membrane vesicles, structures derived from the host endoplasmic reticulum membranes [7] where the duplication and transcription of viral genomes are thought to take place [8]. We decided to show several possible mechanisms in parallel at a subcellular rather than the molecular level to better reflect the current state of knowledge. The viral transcription mechanism was another topic where the research community had yet to reach a consensus at the time of creating the animation. We identified 2 conflicting hypotheses through literature reviews [9] and discussions with experts and decided to visualize these independently through 2 separate animations. In addition to these areas of significant uncertainty, we also had to fill other knowledge gaps to create a complete animation. For example, the depicted mechanisms of polyprotein processing and insertion of nonstructural proteins into endoplasmic reticulum membranes had not been clearly described in the literature and should be considered speculative. Also, while some molecular structures used in the animation were experimentally solved, others represent computational predictions.

## An accompanying interface that supports annotation and scientific discourse

**Fig 2:**
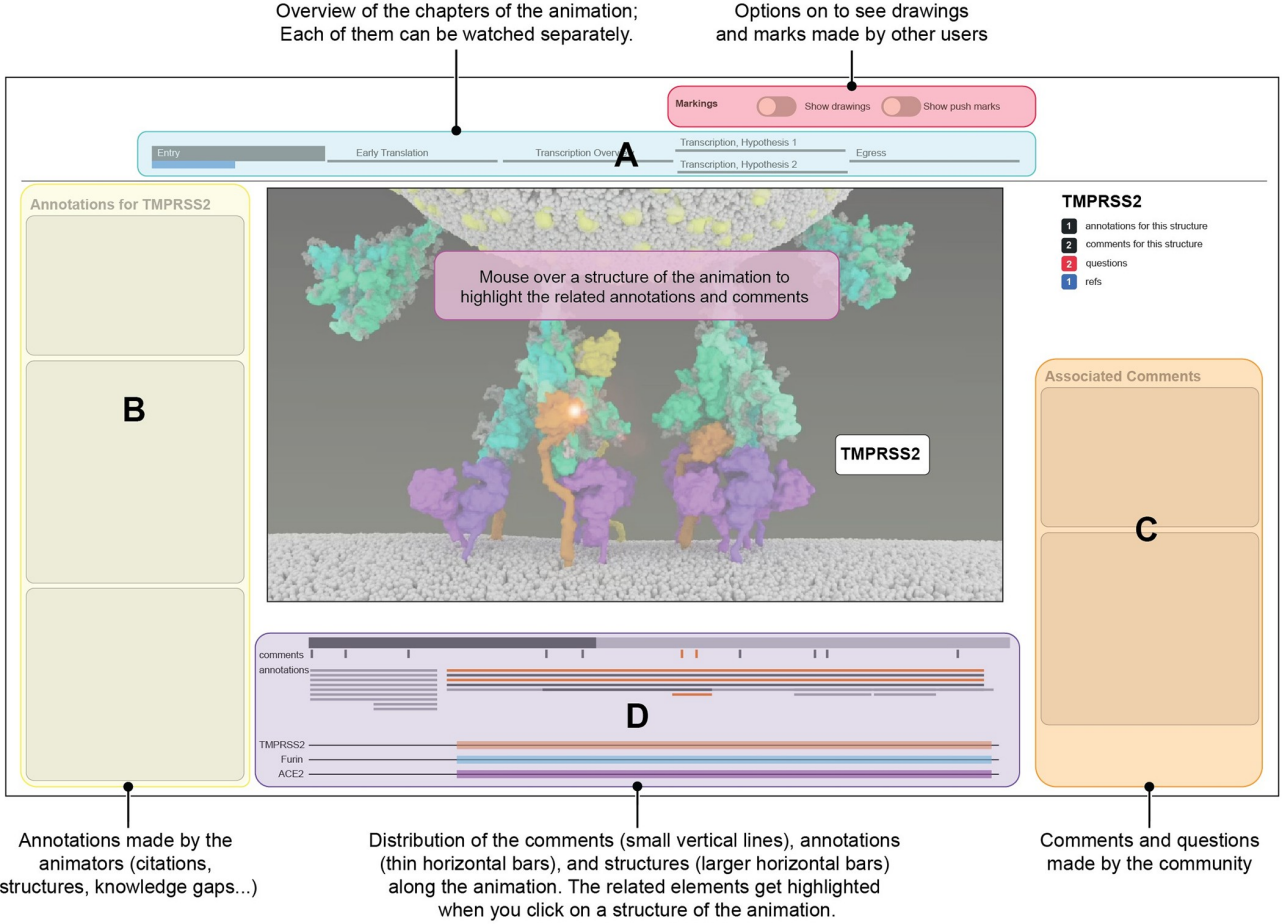
Key capabilities and features of the web-based annotation and commenting tool. These include in particular a time outline with branched chapters representing alternative hypotheses that can be viewed independently (A); annotations provided by animators (B); comments and questions the community (C); both annotations and comments are also highlighted in a clear overview (D). Visit https://coronavirus-annotation-3.sci.utah.edu/ to try it yourself!

Deciding how to visually handle uncertainty is inherent to the work of a molecular animator, and in this project, we sought to make those choices and the data we used more explicit to viewers. Concurrent with the development of the life cycle animations, we designed a web-based annotation interface that allows users to interactively click on a molecular structure in the animation and view associated annotations, questions, and comments. Annotations describe the types and sources of data that were used to generate the animations, and the commenting tools enable the research community to discuss different aspects of the animated model. Its main features and capabilities are described in Fig 2, but please visit the website and try it out yourself!

We envisioned that this annotated animation of the SARS-CoV-2 life cycle could be used to promote and share discourse between domain experts and to visualize which aspects of the animated model are less certain. For example, if multiple users make comments at a specific segment of the animation (to make a criticism, ask a question, or make a clarification), this would suggest that this section of the animation likely does not represent a consensus view of the process. These comments also serve as a means for us to understand if additional animations should be made to represent alternative hypotheses, or to update the current animation based on the latest data.

Through this project, which combined molecular animation of the SARS-CoV-2 life cycle and a powerful new prototype annotation tool, we hope to increase the credibility of animation as a means of discourse and communication within the research community.
